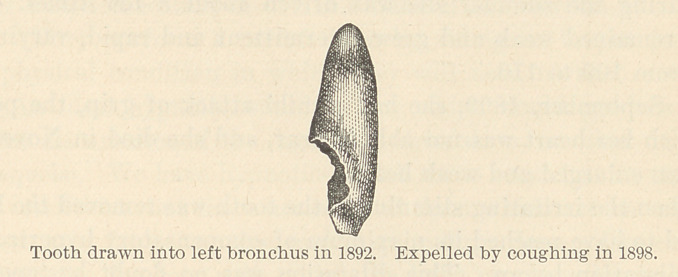# A Case of Swallowing a Tooth

**Published:** 1901-06

**Authors:** G. N. P. Mead

**Affiliations:** Winchester, Mass.


### A CASE OF SWALLOWING A TOOTH.[1]

BY G. N. P. MEAD, M.D., WINCHESTER, MASS.

THE following short history demonstrates the importance of teeth in our physiological economy, quite as much when they are where they should *not* be as when they are where they belong.

Teeth are found in dermoid cysts in the abdomen, they are taken from the œsophagus and stomach by surgeons, and they are normally found in the stomachs of some of the lower orders of animals, as the lobster; but the tooth whose partial history this paper relates was six years in the lung of the patient.

In the physiological lecture-room there used to be told a story by the lecturer—perhaps to test the credulity of his hearers—of an insane man in an institution who was being fed by a soft rubber tube passed through the nose into the stomach, through which soup was poured. One day the tube was passed—let it be hoped by mistake—through the larynx into the trachea and the pint of soup poured into the lungs.

The soup was absorbed and no harm came to the patient. Perhaps if such a custom were persisted in, and coarser food than soup were introduced, nature might evolve teeth in the lungs.

This tooth, however, did not grow in the lung. The patient, Mrs. M. J. R., had had two sisters die of consumption, one at twelve years of age and one at seventy-six years. Her husband died at seventy-nine of heart disease. She had had typhoid fever twice,

---

[1] Read before the American Academy of Dental Science, March 6, 1901.

with a good recovery each time. In May, 1892, she had three teeth of the lower jaw extracted while unconscious from nitrous oxide gas. When she recovered from the gas the dentist was saying, "Hold your head over the bowl, or you will draw blood into your windpipe." She coughed and strangled violently at intervals for two days, when she had a chill, followed by pneumonia and pleurisy of the left side. The pneumonia was protracted for several weeks. She was in bed ten weeks before being able to sit up. It was a year before she could sit up all day. She slowly gained strength enough to drive and to do minor household duties. She was always short of breath, and had copious excretion of a mucopurulent character often stained with blood.

She was seen by me first in September, 1897, five years after the tooth-pulling. She had soreness in the left chest, and was especially weak. The secretion from the lungs amounted to two

Tooth drawn into left bronchus in 1892. Expelled by coughing in 1898.

coffee-cupfuls in twenty-four hours. The character of the secretion was serous, bloody, and slightly purulent. It was raised easily except when she was tired or the lung became more congested than usual.

The appetite and digestion were poor, but she slept well. The respiration was rapid,—thirty to the minute. The pulse was seventy-eight and the temperature normal. A diagnosis of bronchorrhœa was made, but the exciting cause was unknown. The examination of the secretion was negative so far as the bacilli of tuberculosis were concerned. The examination of the chest by inspection showed the left scapula moving somewhat more than the right; percussion showed some dulness at the base of the left lung; and the stethoscope revealed coarse moist râles in the left lung, most marked at the angle of the scapula. There were fine moist râles in the right lung.

She was seen at varying intervals in order to watch the temperature, which proved to be normal, or nearly so, morning and afternoon.

In May, 1898, she had general aches and pains and chilly sensations, with considerable increase in the cough, more difficulty in expectoration, and a temperature varying from 100° to 102° F. She went to bed, and while lying in a horizontal position one morning a week after the beginning of her illness strangled. When the convulsion of coughing was over she found the tooth in her mouth.

Six years the lung had been trying to expel the intruder. The patient quickly rallied from the grip attack, but the pulse was weak and thready in contrast to the strong, full quality which had always characterized it.

The expectoration diminished to one-eighth the quantity, and the blood disappeared. The right lung cleared and the respiration was less rapid, varying from twenty to twenty-eight a minute.

During the summer she was driven about a few times. Her pulse remained weak and grew intermittent and rapid, varying in rate from 100 to 110.

In September, 1899, she had a mild attack of grip, the poison of which her heart was not able to bear, and she died in November from an enlarged and weak heart.

When the irritating stimulus of the tooth was removed the heart seemed to have reached its maximum of compensatory hypertrophy, and dilatation began. This dilatation was no doubt hastened by the attack of grip, her weight, which was about one hundred and eighty pounds, and her age, which was seventy-five.

The literature of the Boston Medical Library was thoroughly searched, and only sixteen cases besides the one reported were found of teeth drawn into the trachea below the larynx; of these, seven were fatal and eight recovered, either by coughing the tooth through the larynx or through an opening made by the surgeon in the trachea below the larynx. The longest time teeth have remained in the lung is thirteen years. The case was reported by W. G. Carpenter, in Guy's Hospital Reports, London, 1842. The patient died of pleurisy, and a piece of plate with four artificial teeth was found in the right pleural cavity, having ulcerated there from the left bronchus.

The longest time teeth have remained in the lung with the recovery of the tooth and patient, except the case reported to-night, is six months. This case was reported by G. A. Himmelsbach in the *Buffalo Medical Journal* for August, 1897. The tooth was a molar coughed spontaneously through the vocal slit.

Therefore this tooth has remained in the lung longer than any case reported in literature in which the patient survived the recovery of the tooth.

## Figures and Tables

**Figure f1:**